# Simultaneous total robotic right hemicolectomy and right partial nephrectomy

**DOI:** 10.1093/jscr/rjad434

**Published:** 2023-07-29

**Authors:** Yohei Sanmoto, Takayuki Hosoi, Shunji Kinuta

**Affiliations:** Department of Surgery, Takeda General Hospital, Fukushima, Japan; Department of Urology, Takeda General Hospital, Fukushima, Japan; Department of Surgery, Takeda General Hospital, Fukushima, Japan

## Abstract

The incidence of synchronous colorectal and renal cancers is reportedly as low as 0.33%. Simultaneous surgery for multi-organ cancers has been reported to have several advantages if tolerated by the patient. In addition, robotic surgery has gained wide application in various fields, but few reports exist on total robotic surgery involving multiple organ resections. We performed simultaneous total robotic surgery on a patient with combined colorectal and renal cancers. Before surgery, we examined the procedure with the surgical team, shared a portion of the trocar site without impairing the operability of the robotic surgery and performed the surgery safely. Further examinations are required to standardize the procedure for simultaneous robotic surgery for multi-organ cancers.

## INTRODUCTION

Synchronous primary malignancies are reported to represent 1% of cancer cases [1]. Conventionally, when cancers are present in multiple organs, and simultaneous surgery is possible, various specialist surgeons cooperate to perform a laparotomy or endoscopic surgery. Simultaneous surgery has several advantages, such as not delaying treatment of remaining cancer, starting postoperative adjuvant chemotherapy sooner if necessary and avoiding a second anesthetic procedure [2–4]. Nowadays, robotic surgery is gaining popularity and is being performed in various fields. However, few reports exist on simultaneous robotic surgery across multiple organs, and standard surgical procedures are lacking. Here, we report a challenging approach to simultaneous total robotic right hemicolectomy and right partial nephrectomy.

## CASE REPORT

**Figure 1:**
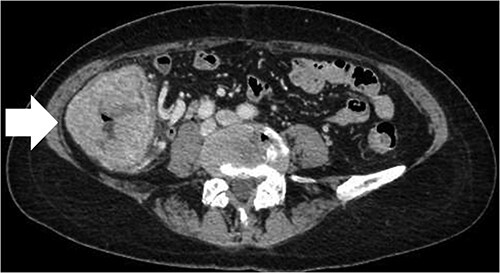
CT scan of the large tumor in the ascending colon (arrow).

**Figure 2:**
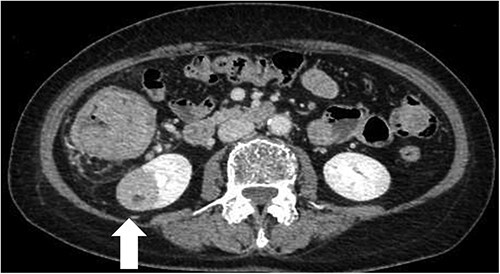
CT scan of the renal tumor in the lower pole of the right kidney (arrow).

**Figure 3:**
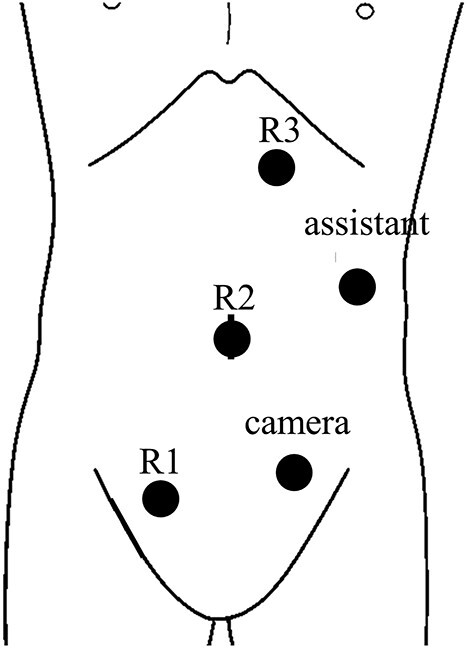
Trocar placement for robotic right hemicolectomy with the patient in the supine position.

A 68-year-old female patient presented to our hospital with fatigue. She had a history of hypertension and hyperlipidemia. She appeared pale, but a physical examination revealed no other abnormalities. Laboratory findings revealed severe anemia, with a hemoglobin level of 8.8 g/dl. Abdominal computed tomography (CT) revealed tumors in the ascending colon and right lower kidney (Figs 1 and 2). Colonoscopy confirmed a Type 1 tumor in the ascending colon, and biopsies of this lesion revealed papillary adenocarcinoma. Preoperative TNM classification according to UICC classification was colon cancer, stage cT3N0M0, and right renal cancer, stage cT1aN0M0. Because both lesions were indications for robotic surgery, the patient underwent synchronous robotic right hemicolectomy and right partial nephrectomy using the robot da Vinci® Xi. Following the induction of general anesthesia, the patient was placed in the supine position. The first 8-mm trocar was inserted through the umbilicus using the Hasson method. The pneumoperitoneal pressure was maintained at 12 mmHg. Additionally, three 8-mm trocars were inserted under laparoscopic vision in the right lower abdomen, left lower abdomen and upper left abdomen, and a 12-mm trocar for assistance was inserted in the left abdomen (Fig. 3). The table was rotated slightly to the left and 10° head down. The robot was rolled in from the patient’s right side and docked. Robotic Arm 1 was affixed with bipolar forceps, Arm 2 had monopolar scissors and Arm 3 had a cadiere forceps. Starting with the inferior approach, the right ureter, gonadal vessels and Gerota’s fascia were preserved. The duodenum and pancreatic head were spared on the dorsal side, and the mesentery was mobilized.

**Figure 4:**
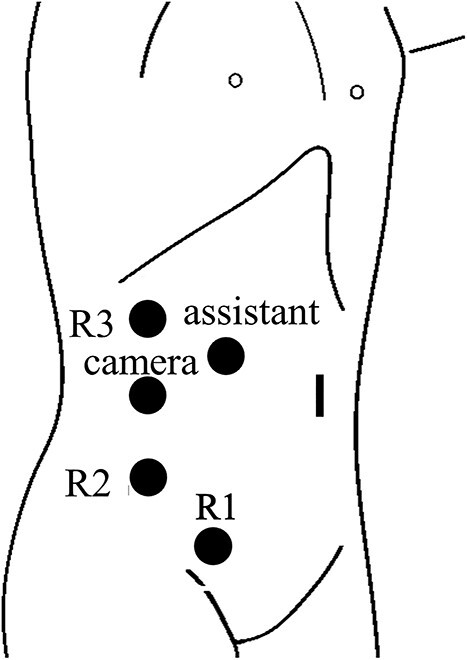
Trocar placement for robotic right partial nephrectomy with the patient in the left flank position. R1, located in right lower abdomen, was shared with the gastroenterologists.

After switching to the medial approach, the ileocolic vessels and the right branch of the middle colic vessels were dissected and secured using a Hem-o-lok^®^ clip. The hepatic flexure was removed from the lateral and cranial sides, and the right hemicolon was completely mobilized from the Toldt fascia. After undocking, the trocar sites were temporarily closed. The patient was then placed in the left flank position. Reusing the port site on the right lower abdomen, three additional 8 mm trocars and one 12 mm trocar for assistance were inserted (Fig. 4). The robot was then redocked to the patient’s right side. Partial nephrectomy was performed by urologists using the arterial clamp technique. The kidney specimens were then placed in an endobag. The robot was undocked, and the trocar sites were completely closed. The warm ischemic time was 15 min. The patient was placed in the supine position again. The gastroenterologists resumed the surgery. The umbilical incision was extended up to 4 cm and protected by a wound retractor^®^. The kidney specimen and right hemicolon were removed, and lymph node dissection was performed. The bowel was transected with a 60-mm linear stapler, Echelon^®^, and a functional end-to-end anastomosis was performed. A suction drain was laparoscopically placed under the liver. During the surgery, three postural changes and two dockings were required. The total operative time was 328 min: 140 min for right hemicolectomy without bowel resection, 118 min for partial nephrectomy and 70 min for bowel resection, anastomosis and fascia closure. The estimated blood loss was 30 ml. The colon with ligated vessels was intraperitoneally for ~2 h, but lactate was within normal range after the operation. The patient’s postoperative course was uneventful. The patient resumed eating on postoperative Day 3. The drain was removed on postoperative Day 6. The patient was discharged on postoperative Day 11. The pathological diagnoses were papillary adenocarcinoma of the ascending colon, stage pT3N0M0, and chromophobe renal cell carcinoma of the right kidney, stage pT1aN0M0. The patient remained cancer-free with no evidence of recurrence 6 months after surgery.

## DISCUSSION

The incidence of synchronous colorectal and renal cancer is reported to be as low as 0.33%, although it is heterogeneous depending on the report [5]. The heterogeneity occurs because of the different prevalences of each cancer [6]. Simultaneous surgery prolongs the operative time but has numerous advantages if the patient can tolerate it. The advantages include decreased hospital stay, less postoperative pain and morbidity, early return to work and avoidance of a second anesthetic procedure [4, 7]. In addition, robotic surgery is generally said to be expensive, but simultaneous surgery has the advantage of reducing costs by sharing the instruments. From a cancer treatment perspective, simultaneous surgery enables early adjuvant chemotherapy for advanced cancer patients compared to when two procedures are performed separately [3]. Regarding surgical safety, adhesions in the operative field may become a problem in multiple surgeries, but their effect can be ignored in simultaneous surgeries. In particular, when the two operations share the same surgical procedure, the advantage of simultaneous surgery seems to be great [8]. In this case, the gastroenterologists performed the right hemicolon mobilization first so that a comfortable operative field was secured when the urologists performed the partial nephrectomy. Trocar placement was significantly different between the right hemicolectomy and partial nephrectomy; however, the unused trocar site was closed with temporary suturing of the epidermis, maintaining airtightness and no subcutaneous emphysema. For this simultaneous surgery, it was essential to discuss and share the surgical procedure, trocar placement and patient position with the gastroenterologists, urologists and operating room team before the surgery. Careful preoperative preparation allowed the operation to be performed safely without compromising the efficiency of the robotic surgery.

## Data Availability

Date sharing is not applicable to this article as no new data were created or analyzed in this study.
